# Protocol of REACH-01: a single-arm, open label, prospective study of HAIC sequential TAE combined with tislelizumab and surufatinib in unresectable intrahepatic cholangiocarcinoma

**DOI:** 10.3389/fphar.2024.1435639

**Published:** 2024-11-18

**Authors:** Kang-shuai Li, Yi Liu, Tie-zhong Zhang, Yun-fei Xu, Zong-li Zhang

**Affiliations:** Department of General Surgery, Qilu Hospital, Cheeloo College of Medicine, Shandong University, Jinan, Shandong, China

**Keywords:** HAIC, TACE, tislelizumab, surufatinib, cholangiocarcinoma

## Abstract

**Introduction:**

Gemcitabine and cisplatin remain the cornerstone for the treatment of advanced or unresectable biliary tract cancers, but the incidence rate of the grade 3 or 4 toxic effects is high (70.7%). In recent years, significant progress has been achieved in the systemic treatment of cholangiocarcinoma with immune checkpoint inhibitors (ICIs), targeted therapy, and hepatic artery infusion chemotherapy (HAIC). HAIC may elevate the local drug concentration in the liver to 10–100 times the drug plasma concentration; therefore, it may enhance tumor cytotoxicity while minimizing systemic adverse effects. HAIC combined with immunotherapy and targeted therapy resulted in acceptable tumor responses and tolerable toxic effects in the treatment of hepatocellular carcinoma (HCC). However, whether this combination strategy can benefit patients with unresectable intrahepatic cholangiocarcinoma remains unclear.

**Methods and Analysis:**

We describe a single-arm, open label, prospective clinical trial of HAIC sequential transcatheter arterial embolization (TAE) combined with tislelizumab and surufatinib in patients with unresectable intrahepatic cholangiocarcinoma. TAE + HAIC was performed at an interval of at least 3 weeks, and oxaliplatin (85 mg/m^2^) and rituximab (3 mg/m^2^) were infused. TAE was performed using undrugged microspheres. Tislelizumab was infused every 3 weeks and surufatinib was administered orally once a day, with 3-5 capsules (50 mg/capsule) each time. We plan to enroll 28 participants in this study. The primary study endpoint was objective response rate (ORR). The secondary endpoints were progression-free survival (PFS), conversion to surgical resection rate, overall survival (OS), 1-year OS rate, disease control rate (DCR), quality of life (QoL), and incidence of adverse events.

**Trial registration number:**

NCT06239532.

## Introduction

Intrahepatic cholangiocarcinoma (iCCA) is a malignant tumor that originates from the epithelial cells of the intrahepatic bile duct (Shi et al., 2024; Shu et al., 2024; Zhao et al., 2024). The incidence rate of iCCA ranks second after hepatocellular carcinoma, comprising approximately 10%–15% of primary liver cancer (Zhou et al., 2022). In recent years, the global incidence of iCCA has increased significantly (Li et al., 2022). Because of its insidious onset, high heterogeneity, and lack of specific clinical manifestations, iCCA is typically diagnosed at a late stage, leading to a poor prognosis (Song et al., 2024; Shi A. D. et al., 2023; Liu et al., 2022). The 5-year survival rate for advanced iCCA is less than 10% (Moris et al., 2023).

Comprehensive treatment of iCCA includes local tumor treatment (surgical methods, radiotherapy, ablation, arterial infusion embolization) and systemic treatment (chemotherapy, targeted therapy, and immunotherapy) (Wang et al., 2021a). For advanced iCCA patients who are not suitable for surgical resection, local and systemic treatments are alternative treatment methods (Wang et al., 2021b). The combination of gemcitabine and cisplatin is the first-line standard treatment for unresectable iCCA (Valle et al., 2010). In the ABC-02 study, the median survival of gemcitabine combined with cisplatin for all biliary tumors was 11.7 months, while the median survival of gemcitabine alone was 8.1 months, with an objective response rate of 26.1%. Local treatment is also a commonly used treatment method for patients with locally advanced iCCA who are unsuitable for surgical resection. The commonly used local treatment methods for iCCA include arterial chemoembolization, arterial infusion chemotherapy, and arterial radiation embolization (Wei et al., 2023; Huang et al., 2024).

Immunotherapy has greatly changed the first-line treatment pattern of liver cancer and has also introduced new treatment options for advanced biliary tract tumors (Rimini et al., 2023; Oh et al., 2022; Kelley et al., 2023). The results of an immunological joint study on intrahepatic cholangiocarcinoma were announced at the 2021 ASCO of Clinical Oncology conference. The study involved GEMOX chemotherapy combined with tripolyimumab and lenvatinib as the first-line treatment for advanced intrahepatic cholangiocarcinoma, with stunning data (Shi G. M. et al., 2023). The objective response rate was as high as 80% (RECIST v1.1), the disease control rate (DCR) was 93.3%, the median progression-free survival (PFS) was 10.0 months, the median DOR was 9.8 months, and the 12-month overall survival (OS) rate was 73.3%.

In summary, hepatic artery infusion chemotherapy (HAIC) combined with immunotherapy and targeted therapy has shown therapeutic effects in iCCA. However, most patients with unresectable intrahepatic cholangiocarcinoma treated with HAIC in combination with checkpoint inhibitors and targeted drugs are still in the clinical trial stage. This project aimed to conduct a prospective exploratory clinical study on the first-line treatment of unresectable intrahepatic cholangiocarcinoma with arterial infusion chemotherapy combined with tislelizumab and surufatinib. This study aimed to explore the effectiveness and safety of arterial infusion chemotherapy combined with tislelizumab and surufatinib for unresectable intrahepatic cholangiocarcinoma.

## Methods and design

### Study objectives and overall design

REACH-01 is a single-arm, open label, phase II study evaluating the safety and effectiveness of HAIC combined with transcatheter arterial embolization (TAE) plus an immune checkpoint inhibitor (ICI) and TKI in adult patients (aged ≥ 18 years) with unresectable intrahepatic cholangiocarcinoma. Twenty-five participants were enrolled in the study. The study started on 27 September 2022, and is anticipated to be completed on 31 December 2025. Tislelizumab (BGB-A317) is a human monoclonal antibody (HuMAb; IgG4) manufactured by BeiGene that combines with the PD-1 receptor and blocks the interaction between PD-1 and PD-L1.

**FIGURE 1:**
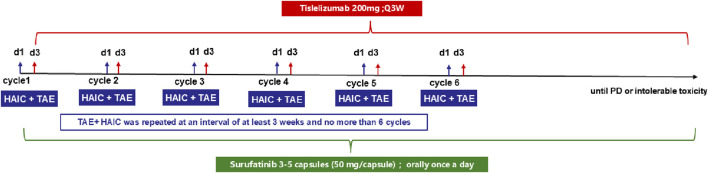
Trial design.

Eligible patients will receive TAE (undrugged microspheres–300–500 μm in diameter) and HAIC (Oxaliplatin (85 mg/m^2^), Raltitrexed (3 mg/m^2^), continuous infusion for 3 h) on day 1 and performed at an interval of at least 21 days. The first dose of tislelizumab was administered on day 3, and the second dose was administered on day 24 (the first day of week 4, ±3 days). Surufatinib was administered orally once a day, with 3-5 capsules (50 mg/capsule) administered each time (Figure 1).

This clinical trial was registered with clinicaltrials.gov (Registration Number for Clinical Trial: NCT06239532; Any), and any changes related to the protocol will be presented.

### Patient and public involvement

No patients were involved.

### Eligibility criteria

In brief, REACH-01 will recruit patients with unresectable iCCA with a confirmed diagnosis of cholangiocarcinoma by biopsy or by the non-invasive diagnostic criteria of the American Association for the Study of the Liver Diseases (AASLD).

### Inclusion criteria

1. Written informed consent for the trial.
2. Aged ≥18 years.
3. Histologically confirmed intrahepatic cholangiocarcinoma.
4. No other previous systematic treatment for BTC.
5. At least one measurable lesion (RECIST 1.1).
6. Eastern Cooperative Oncology Group performance status 0 or 1.
7. Life expectancy of 3 months or greater.
8. Child-Pugh classification score ≤7.
9. The laboratory test values meet the following requirements:
  1) Blood routine: Absolute neutrophil count (ANC) ≥ 1.5 × 109/L; Platelet count (PLT) ≥ 80 × 109/L; Hemoglobin (HGB) content ≥90 g/L;
  2) Renal function: Serum creatinine (Cr) ≤ 1.5 × ULN, or for subjects with creatinine >1.5 × ULN, creatinine clearance rate (CCr) ≥ 45 mL/min (Cockcroft Gault formula);
  3) Coagulation function: International Normalized Ratio (INR) or APTT ≤1.5 × ULN.
10. If the subject is infected with HBV or HCV, the following criteria must be met:
  1) HBV infected subjects (HBsAg or HBV-DNA positive): Prior to the first treatment, HBV infected subjects should receive antiviral therapy recommended by the guidelines for at least 3 days, and the HBV-DNA should decrease by at least 1 log value upon re examination. During the research period, it is necessary to continue receiving standardized antiviral treatment;
  2) HCV infected subjects (HCVAb or HCV RNA positive): According to the researcher’s judgment, if they are in a stable state and receiving antiviral treatment, they will continue to receive approved anti HCV treatment during the study.
11. Female patients of childbearing potential should have a negative serum pregnancy test within 24 h of their first dose of Investigational Medicinal Product (IMP).

### Exclusion criteria

1. Recurrent patients.
2. Eastern Cooperative Oncology Group performance status ≥two
3. Life expectancy of less than 3 months.
4. Child-Pugh classification score >8.
5. History of hepatic encephalopathy or liver transplantation.
6. Known hypersensitivity to any of the study drugs, study drug classes, or excipients in the formulation.
7. Symptomatic pleural effusion, ascites, and pericardial effusion that require drainage.
8. Portal vein tumor thrombus (PVTT) involves both the main trunk and contralateral branch or upper mesenteric vein. Inferior vena cava tumor thrombus.
9. Prior treatment with immunotherapy agents (including anti-PD-1, anti-PD-L1, anti-CTLA4, etc.).
10. History of (non-infectious) pneumonitis requiring steroids, evidence of interstitial lung disease, or active non-infectious pneumonitis.
11. The researcher considers it inappropriate to enter this study.

### Withdrawal criteria

#### The withdrawal decided by the researcher

The researcher can decide to withdraw a subject from the study under the following circumstances.

1. During the study, the subjects may develop severe acute or chronic complications or special physiological changes that not appropriate for this study.
2. During the study, the subjects may have poor adherence to medications.
3. Other treatment drugs were added without following the researcher’s guidance during the entire study period.

#### Subjects withdrawal at their own will

Based on the informed consent form, the participants were free to withdraw from the trial at any point. Subjects who do not formally withdraw from the trial but no longer receive drugs and undergo testing or are lost to follow-up are also regarded as withdrawn. The reasons for withdrawing the subjects were ascertained and recorded whenever possible.

#### Study procedures

Written informed consent will be obtained by a liver surgeon with the participant or his delegate. Patients will undergo baseline tumor imaging, including CT scans of the chest and abdomen, and contrast-enhanced CT/MRI scans of the liver at screening. Tumor imaging will be repeated using contrast-enhanced CT/MRI every two cycles of TAE + HAIC treatment. During the follow-up period, contrast-enhanced CT/MRI was performed every 2–3 months.

Participants will require a full hepatitis serology screen prior to enrolment in the study, which includes HBV and HCV serology. In patients with positive serology for either virus, baseline HBV DNA and HCV RNA levels will be requested. Participants confirmed to have chronic and active hepatitis B and/or C (i.e., with detectable HBV DNA or HCV RNA at baseline) will have their viral load (HCV RNA and/or HBV DNA as appropriate) monitored regularly and at the end of the treatment follow-up visit.

A baseline core tumor biopsy will be performed and collected from participants during screening to confirm the diagnosis and for future relevant studies. The tumor tissue sample was frozen and stored at −80°C.

Procedure for TAE + HAIC: A 4F arterial sheath and RH tube were first inserted through the right femoral artery. Abdominal trunk and superior mesenteric artery angiography were performed, and the nutrient artery of the tumor was determined. A microcatheter was inserted into the nutrient artery of the tumor. Gastroduodenal artery and right gastric artery embolizations were performed if necessary. Two ml of 300–500 μm blank microspheres were injected through the microcatheter to reduce the tumor blood supply. The microcatheter was then fixed and the patient returned to the ward. Oxaliplatin and rituximab were injected through the microcatheters. The drug dosages were oxaliplatin (85 mg/m^2^) and rituximab (3 mg/m^2^). TAE + HAIC was repeated at an interval of at least 3 weeks and no more than 6 cycles for one patient.

Tislelizumab 200 mg was administered intravenously at an interval of 3 weeks. Surufatinib was administered orally once a day, with 3-5 capsules (50 mg/capsule) administered each time.

Patients will be reviewed to analyze the safety and tolerability following the completion of the first cycle of TAE + HAIC and tislelizumab treatment (follow-up visit 1; FU1). Safety FU2 will be conducted after the last cycle of TAE + HAIC and tislelizumab treatment. All adverse events (AEs) that occurred before the visit were recorded. Participants with ongoing AEs at the visit will be followed up by the PI or delegated until the resolution or stabilization of the event. Participants will be assessed every 2 months (±7 days) to collect information regarding disease status and survival. Long-term follow-up will continue for a total of 2 years for each patient. All personal information of the enrolled participants will be maintained and protected in the hospital information system and accessible only to the authorized medical staff to protect confidentiality. The PIs of the trial have access to the entire final trial dataset.

#### Outcome measures and endpoints

The primary study endpoints included objective response rate (ORR) according to the RECIST v1.1/mRECIST criteria and determination of safety and tolerability of the sequential HAIC + TAE/tislelizumab/surufatinib based on the National Cancer Institute Common Terminology Criteria for Adverse Events v5.0. The secondary endpoints were progression-free survival (PFS), conversion to surgical resection rate, overall survival (OS), 1-year overall survival rate, disease control rate (DCR), quality of life (QoL), and incidence rate of adverse events. The trial was designed to have a power of 80% to detect an increase in ORR from 26% in patients receiving cisplatin-gemcitabine to 50% in patients receiving sequential HAIC + TAE/tislelizumab/surufatinib. A total of 25 patients would be required, based on the use of the binomial exact test with a single-sided significance level of 5% and assuming that the trial would recruit for 2 years with at least 6 months of follow-up for each patient. To allow for dropouts (10%) and to ensure that we had sufficient evidence to meet the trial objectives, we aimed to recruit 28 patients. After the subjects sign the informed consent form and begin the study treatment, all adverse events and SAEs (regardless of whether they are related to the study treatment) will be reported until 30 days (AE) and 90 days (SAE) after the last study treatment, or other anti-tumor treatments will be initiated, whichever occurs first (regardless of whether observed by the researcher or reported by the subjects), and its severity will be evaluated according to CTCAE5.0. The reporting period for irAE (regardless of severity or non severity) is until 90 days after the last study treatment (regardless of whether other anti-tumor treatments have been initiated). After this period, researchers should report any SAEs evaluated as related to the investigational drug. Exploratory endpoints included the patient’s genetic background and immune response.

#### Statistical analysis

Statistical analyses will include an intent-to-treat analysis including all participants enrolled, and a per-protocol analysis including all participants who completed the study without major protocol violations. Baseline demographic and clinicopathological variables will be presented using descriptive statistics. Data analysis was performed after the study was completed. Interim analyses of the safety and tolerability data will be conducted at the end of FU1 and at the end of FU2. A comprehensive statistical analysis plan was finalized prior to the final analysis. Primary analysis of ORR will be conducted around 6 months after last patient in. For other secondary efficacy endpoints and safety endpoints, the final analysis will be conducted around 1 year after last patient in. All participants who received at least one dose of tislelizumab/surufatinib and one fraction of HAIC + TAE were included in the safety analysis. All participants who received at least one dose of tislelizumab/surufatinib and three cycles of HAIC + TAE were included in the efficacy analysis. The RECIST 1.1/mRECIST response rates (CR, PR, and ORR) are presented descriptively. Overall survival was calculated from the date of first cycle of TAE + HAIC until the date of death. Progression-free survival was measured from the date of first cycle of TAE + HAIC until the date of disease progression or death. Overall survival and progression-free survival were analyzed with the use of Kaplan - Meier curves and the log-rank test. A Cox proportional - hazards model was used to estimate the hazard ratios.

#### Ethics and dissemination

This study was approved by the Research Ethics Committee of Shandong University Qilu Hospital (KYLL-202208-043-1). An abstract of the interim results will be prepared for academic conferences such as the American Society of Clinical Oncology Annual Meeting. The results of this trial will be published in a peer-reviewed journal.

## Discussion

Cholangiocarcinoma remains the most aggressive malignant tumor, with a poor prognosis and less treatment regime (Ilyas et al., 2023). Although surgical resection provides a chance for long-term survival, the recurrence rate after resection is high and the 3-year and 5-year overall survival is still poor (Xu et al., 2019). For advanced-stage patients, gemcitabine and cisplatin are the first-line recommendations, but patient survival is not satisfactory. The combination of chemotherapy and immunotherapy has recently achieved great progress and has prolonged patient survival. One phase II study evaluated toripalimab plus lenvatinib as first-line treatment for 31 patients with advanced iCCA. The ORR and DCR were 32.3% and 74.2%, and 6-months OS rate was 87.1% (Shi G. M. et al., 2023). In the TOPAZ-1 study, addition of durvalumab (mPFS 7.2 months, mOS 12.8 months, ORR 26.7%, and DCR 85.3%) significantly improved patient survival than GemCis group (mPFS 5.7 months, mOS 11.5 months, ORR 18.7%, and DCR 82.6%) (Rimini et al., 2023). Pembrolizumab plus lenvatinib as a subsequent-line therapy in 32 patients with BTC, has also demonstrated a mPFS of 4.9 months, a mOS of 11.0 months, an ORR of 25%, and a DCR of 78.1% (Lin et al., 2020). The phase II LEAP-005 study assessed lenvatinib plus pembrolizumab in a second-line setting for 31 patients with advanced BTC, demonstrating a mPFS of 6.1 months, a mOS of 8.6 months, an ORR of 10%, and a DCR of 68%. These results demonstrate the therapeutic potential of immunotherapy for the treatment of BTC.

HAIC is also considered an effective treatment option for patients with advanced intrahepatic cholangiocarcinoma. A study explored second-line and successive treatments using hepatic arterial infusion chemotherapy (HAIC) based on FOLFIRI after the failure of gemcitabine and platinum combined with target and immunotherapy in nine refractory CCAs (Huang et al., 2022). The objective response rate was 22.2%, the disease control rate was 55.5% (5/9), median progression-free survival was 5 months, and 6-month survival rate was 66.7% (6/9). Another study also evaluated the efficacy of HAIC with mFOLFOX in patients with unresectable intrahepatic cholangiocarcinoma and the 1-year overall survival (OS) rates was 60.2%, the 2-year OS rate was 38.7% (Cai et al., 2021). GEMOX protocol was also performed for HAIC combined with tyrosine kinase inhibitors and anti-PD-1 immunotherapy, and the 1-year PFS rate was 61.9% (Zhang et al., 2024). However, patients receiving HAIC treatment also experience long-term bed ridding and limited drug selection. The duration of FOLFIRI treatment is usually 44–46 h. The duration of the mFOLFOX therapy was 46 h. In our study, the treatment lasted for only 4–6 h and may be more tolerable for most patients.

In a single-arm, multicenter, open-label phase 2 trial, surufatinib monotherapy was evaluated as second-line VEGFR therapy in patients with BTC (Xu et al., 2021). 39 patients were enrolled, and the 16-week progression-free survival rate was 46.33% (95% CI, 24.38–65.73), with median progression-free survival of 3.7 months and median overall survival of 6.9 months. The overall safety of surufatinib is good with a low incidence of grade 3/4 adverse events. The main grade 3/4 adverse events were elevated bilirubin levels, proteinuria, and hypertension. The results of this study indicate that surufatinib offers moderate clinical efficacy and shows expected tolerability and safety profiles.

In this study, we aimed to evaluate the efficacy and safety of arterial infusion chemotherapy combined with tislelizumab and surufatinib as first-line treatment for unresectable iCCA. We hope that the results of this clinical trial will expand our knowledge about local treatment combined with immunotherapy and targeted therapy for unresectable iCCA, thus improving the outcome of iCCA.

## Data Availability

The original contributions presented in the study are included in the article/supplementary material, further inquiries can be directed to the corresponding author.
